# Thermal Conduction in Hybrid Nanofluids and Aggregates

**DOI:** 10.3390/nano14030282

**Published:** 2024-01-30

**Authors:** Eugene D. Skouras, Nikolaos P. Karagiannakis, Vasilis N. Burganos

**Affiliations:** 1 Institute of Chemical Engineering Sciences (ICE-HT), Foundation for Research and Technology, Hellas (FORTH), GR-26504 Patras, Greece; eugene.skouras@iceht.forth.gr (E.D.S.); nick_karag@iceht.forth.gr (N.P.K.); 2 Department of Mechanical Engineering, University of the Peloponnese, GR-26334 Patras, Greece

**Keywords:** hybrid nanofluid, heat conduction, effective thermal conductivity, nanoparticle aggregates, aggregate morphology

## Abstract

Hybrid nanofluids contain more than one type of nanoparticle and have shown improved thermofluidic properties compared to more conventional ones that contain a single nanocomponent. Such hybrid systems have been introduced to improve further the thermal and mass transport properties of nanoparticulate systems that affect a multitude of applications. The impact of a second particle type on the effective thermal conductivity of nanofluids is investigated here using the reconstruction of particle configurations and prediction of thermal efficiency with meshless methods, placing emphasis on the role of particle aggregation. An algorithm to obtain particle clusters of the core–shell type is presented as an alternative to random mixing. The method offers rapid, controlled reconstruction of clustered systems with tailored properties, such as the fractal dimension, the average number of particles per aggregate, and the distribution of distinct particle types within the aggregates. The nanoparticle dispersion conditions are found to have a major impact on the thermal properties of hybrid nanofluids. Specifically, the spatial distribution of the two particle types within the aggregates and the shape of the aggregates, as described by their fractal dimension, are shown to affect strongly the conductivity of the nanofluid even at low volume fractions. Cluster configurations made up of a high-conducting core and a low-conducting shell were found to be advantageous for conduction. Low fractal dimension aggregates favored the creation of long continuous pathways across the nanofluid and increased conductivity.

## 1. Introduction

Optimal thermal performance is one of the major objectives in numerous modern applications. Thermophysical features, such as thermal conductivity and specific heat, are key properties of fluids when used in energy applications and affect drastically the overall efficiency. The introduction of nanoparticles into carrier fluids [1,2] improves the thermal performance through the direct increase in the thermal conductivity of the resulting nanofluids. In addition, the fluid viscosity also affects thermal and mass transfer performance through the flow pattern and convective phenomena, not only in energy applications, but also in separation applications using membranes and filters. Similar concepts and efficiency studies apply to the area of nanocomposite materials that contain dispersed phases of nanosized additives or fillers, and are known to exhibit much improved transport and mechanical properties even for low-volume fractions of additives.

Nanofluids are usually prepared in a two-step process. Typically, nanoparticle production is performed first, followed by particle suspension in a base fluid [3]. Commercial nanoparticles usually come in powder form. Nanoparticle fabrication steps usually include the creation of a nanoparticle suspension from precursors, which is dried using various methods to obtain a powder [4,5]. Solid nanoparticles can also be produced through thermal decomposition of organic precursors [5]. In addition, nanoparticle fabrication can be performed in the gas phase [6,7], following some key formation mechanisms, among which are condensation, coagulation, and surface reactions [7,8,9,10]. The kinetics of each process determines the final structural morphology, which can vary among, and most typically are spherical particles, agglomerates, or compact aggregates [9].

To improve further the thermal properties and alleviate dynamic viscosity increase in nanoparticulate systems, while also ensuring cost-effective production, hybrid nanofluids have been introduced [11,12]. A nanofluid is considered hybrid when its constituent nanoparticles include more than one distinct species. Hybrid nanofluids have recently attracted strong attention as promising heat-transfer fluids [11,13]. The improvement of the thermal characteristics of hybrid nanofluids is usually attributed, among others, to improved thermal pathways, the synergistic effect of the constituent species, and more efficient shapes [14]. Hybrid materials are expected to combine the thermophysical properties of two or more nanoparticulate materials in a synergistic fashion when combined in a mixed phase rather than in single-particle-type nanofluids [14]. This is also confirmed by a literature survey [11,13,14,15]. However, a number of studies have claimed that the thermal performance of certain hybrid nanofluids can be lower than single-component ones. This underperformance is ascribed to inappropriate relative size of the nanomaterials used, incompatibilities of the nanoparticles with each other, and varying degree of stability of the resulting nanofluid depending on temperature [11,15]. Thus, it is necessary to consider several aspects of the process in order to guide the fabrication of a hybrid nanofluid with the desirable performance.

Many studies have focused on the prediction of the heat transfer properties of nanofluids, however with no broadly accepted justification of their performance or a reproducible approach to predict heat conduction properties [16,17]. The effects of the interfacial resistance, Brownian motion of the nanoparticles, and the presence of nanolayers around the particles, including that of surfactants, as well as aggregation effects, have been studied methodically by the authors and other investigators [18,19,20,21,22,23]. The volume fraction of the nanoparticles is typically considered as a key component that greatly influences the performance of nanofluids. It has been shown, in general, that an increase in the volume fraction of dispersed nanoparticulates causes improvement in the thermal conductivity and modification of the dynamic viscosity of the fluid, as stated by several works on nanofluids [24,25,26].

A notable increase in the thermal conduction coefficient has also been reported experimentally if the nanoparticles are organized in small aggregates [27,28]. The thermal bridge between particles that are in contact within an aggregate was found to facilitate heat transport compared to fully dispersed nanoparticles. On the contrary, aggregates consisting of a large number of particles are usually related to decreased stability and increased viscosity of the nanofluid, thus demoting nanofluid properties [29].

In the present work, the effects of multiple particle types on the effective thermal conductivity of nanofluids that contain particle aggregates are examined. To this end, the method that was developed by the authors [22,23] for reconstructing particle aggregates is extended to include hybrid nanoparticulate configurations. This method offers rapid stochastic reconstruction of agglomerated systems with tailored properties, including, most notably, the desired fractal dimension and the average number of particles per aggregate. The effective thermal conductivity is estimated through the temperature profiles that are calculated from the solution of the heat transport equation in the solid particle domain and the carrier fluid domain, complemented by appropriate boundary conditions. The Meshless Local Petrov–Galerkin (MLPG) method has been shown to provide stable and fast solutions to thermofluidic systems that contain particles in contact [30,31,32], and is utilized for the solution of the heat transport equations. The Discretization-Corrected Particle Strength Exchange (DC PSE) method [33,34] is used to approach the field function and its derivatives. The meshless nature of the method allows local increase in the domain discretization at the interface between the base fluid and the solid particles. The aforementioned numerical technique is employed here to handle the well-known complication that arises at particle contacts, which usually becomes prohibitive to accurate and reliable solutions. In fact, this method is adapted here to be able to treat large nanoparticle systems and ensure statistically meaningful results.

The effects of a second particle component that exists in a hybrid nanofluid and the impact of cross-aggregation of the different particle types on the effective thermal conductivity of the nanofluid are investigated in the present work. The study includes variation of the number of particles in the aggregates, the fractal dimension of the aggregates, the volume fraction of the particles, and the mixing ratio of the two sets of nanoparticles and attempts to elucidate the role of these factors in the overall heat transfer behavior of the nanofluid. The impact of the aggregation degree on the effective conductivity of the hybrid nanofluids, as predicted by the present method, is compared to the corresponding impact from experimental measurements for the same or similar characteristics. Moreover, the effective conductivity of hybrid nanofluids containing aggregated particles is compared to that of single-particle-type suspensions, keeping all other morphological parameters constant. The results are also compared with predictions of analytic expressions, such as effective medium approximations. A parametric study is carried out to identify those parameter values that appear to improve the performance of the hybrid nanosystems, placing emphasis on the aggregation degree and aggregate configuration features.

## 2. Simulation of Hybrid Nanofluids and Mixed-Type Aggregates

The approach that is undertaken in this work is that the thermal properties of hybrid fractal aggregates can be correlated with their morphological parameters through digital reconstruction of their representative configuration and numerical computation of their thermal conductivity. Key fabrication factors, such as the physical properties of the nanoparticles and the base fluid, the particle shape and dispersion level, and temperature are known to influence the particle aggregation process drastically [20,21]. Stochastic particle aggregate methods, such as Diffusion-limited Aggregation (DLA), Diffusion-limited Cluster-Cluster Aggregation (DLCCA), Reaction-limited Aggregation (RLA), and Ballistic Aggregation (BA), have been implemented for the computational generation of fractal structures [34,35], with varying levels of success when compared to experimental data [36,37]. A collective description of said aggregation methods is the fractal dimension, $d_{f}$, that quantifies the structural morphology and complexity through an expression that relates the number of particles in the aggregate, N, with basic cluster-size characteristics,

(1) $$N={k_{g}(R_{g}/r_{p})}^{d_{f}},$$

In Equation (1) $R_{g}$ is the radius of gyration of the aggregate [38], $r_{p}$ is the mean radius of the particles, and $k_{g}$ is the structure factor. The fractal dimension takes values between unity and the working spatial dimension. Aggregates of low fractal dimension tend to spread in a planar or linear form, whereas a high fractal dimension signifies a more spherical distribution of particles [21,39]. The conventional implementation of the above stochastic methods to create aggregates is usually quite expensive computationally. A different method has been proposed by the authors [23,24], that allows fixing the fractal dimension to the desired value and drives the formation of particle aggregates in a stochastic fashion that allows, eventually, reproduction of the target value. This method is particularly fast and shows very promising results for the efficient representation of nanofluids.

In order to study the effect of the co-existence of multiple nanoparticulate species on thermal conductivity, the work developed by the authors in [23] is extended here to include hybrid nanofluids that contain two types of nanoparticles with thermal conductivities *k*_1_ and *k*_2_. The objective is to provide a stochastic representation of aggregate systems with predetermined properties. The key steps of the algorithm are depicted below. The algorithm requires as input the total volume fraction of the particles, the volume fraction ratio of the two components, the fractal dimension, and the average number of particles per aggregate. The random placement of a particle within the working domain initiates the process. A new particle is added on the surface of the deposited particle at a randomly chosen contact point. The process is iterated until each aggregate acquires the predefined number of particles per aggregate, or a predefined distribution of particle numbers per aggregate with a desired mean value. When the desired number of aggregates in the working domain is reproduced, the aggregation simulation is ended. During the entire process, care is exercised to avoid overlapping between any pair of particles that belong to the same or different aggregates. The precise position of each newly inserted particle is guided by the comparison of the current fractal dimension to its desired value [23]. Specifically, increased fractal dimension is most probable to obtain when the new particle is placed in contact with a randomly selected particle within the radius of gyration of the aggregate. The opposite is expected to occur if the new particle is in contact with a particle that lies outside the radius of gyration. This selection process drives the convergence of the fractal dimension to the predefined target value or within a desired range of values. A detailed description of the method for single-particle-type nanofluids is presented in previous work by the authors [23]. The required modifications to account for hybrid configurations are discussed below in this section. For the sake of simplicity in the description of the simulator, the hybrid nanofluid is assumed to consist of uniformly sized particles, i.e., the size of each particle is independent of the particle type. This assumption can be easily removed if sufficient data for the size distributions of the two types of particles are available.

Simulations take place in a cubic box of length l, which is also used as the reference length for the spatial normalization of the model quantities. Thus, the normalized particle radius is related to the total particulate volume fraction (ϕ), the number of particles in each cluster ($N_{p,i}$), and the number of clusters ($N_{c}$), as follows:

(2) $$r_{p}=\sqrt[{3}]{\frac{3\phi }{4\pi \sum_{i}^{N_{c}}N_{p,i}}}.$$

Particles are added sequentially and form aggregates with the desired fractal dimension and the desired number of particles per aggregate, using the procedure that was described above, until the desired total volume fraction is achieved. At this initial stage, no special reference to the type of particles (1 or 2) is made yet. After the aggregates are generated, their particles are labeled as type-1 or type-2 depending on the prescribed mixing ratio. This assignment can be implemented in two different ways: (i) random distribution in the interior of each aggregate, henceforth called Random mixing type, or (ii) placing the particles of type-1 in the outer layer (shell) of the aggregate, and those of type-2 in the kernel (core) of the aggregate, or vice versa, called Core–Shell type. Each of these options is directly relevant to the nanofluid preparation process, namely direct mixing of the two particle populations or sequential dispersion of the two population types.

The Random configuration is implemented in a straightforward manner. After all clusters are generated with the prescribed number of particles, each particle is stochastically assigned the *k*_1_ or the *k*_2_ value based on the population ratio, independently of its position. The Core–Shell configuration is implemented as follows. Initially, all particles are assigned the conductivity (*k_core_*) of the population type that is intended to occupy the core of the aggregates (i.e., *k*_1_ or *k*_2_). An aggregate is randomly chosen and its particles with the lowest coordination number, i.e., with the least contacts with core-type particles, are assigned the conductivity of the second type (*k_shell_*). This process is iterated until the prescribed population ratio is obtained, and repeated for all aggregates. If this ratio is not achieved with the lowest coordination number chosen, an iterative labeling process takes place, as follows. A particle already labeled *k_shell_* is randomly chosen, and its closest neighbor with the second-lowest coordination number, not already labeled *k_shell_*, is chosen and is now labeled *k_shell_*. This process is termed “shrinking core-shell interface process” and is iterated until the target population ratio is obtained.

An outline of the conductivities allocation stage of the algorithm and relevant parameter setting for nanofluids with two distinctive types of particles is as follows. Random configuration step: particles are stochastically sampled from the total number of particles deposited ($N_{c}\cdot N_{p}$), and assigned a conductivity value from the prescribed binary distribution (*k*_1_, *k*_2_) with the given population ratio (*ϕ_p_*). Core–Shell step (i): all ($N_{c}\cdot N_{p}$) deposited particles are assigned the conductivity of the population type assumed at the core of the aggregates (*k_core_*). Core–Shell step (ii): deposited particles are sorted with their coordination number, and the ones with the lowest coordination number (typically unity), generally located at the outskirts of clusters, are stochastically chosen and assigned the second type of conductivity (*k_shell_*), up to the prescribed population ratio (*ϕ_p_*). Core–Shell step (iii): if the prescribed population ratio (*ϕ_p_*) is not achieved with the lowest-coordination-number particles from the previous step, one of them, now labeled *k_shell_*, is statistically chosen and has its neighboring particles in direct contact ranked based on their coordination number. Core–Shell step (iv): Such a neighbor with the second-lowest coordination number, and still labeled *k_core_*, is randomly selected with its label turned to *k_shell_*. This process is iterated with all the second-lowest coordination number neighboring particles up to the prescribed population ratio (*ϕ_p_*). Core–Shell step (v): if the prescribed population ratio (*ϕ_p_*) is not achieved, repetition of Core–Shell steps (iii)–(iv) is performed with the third (and so on) lowest coordination number neighbors.

**Figure 1:**
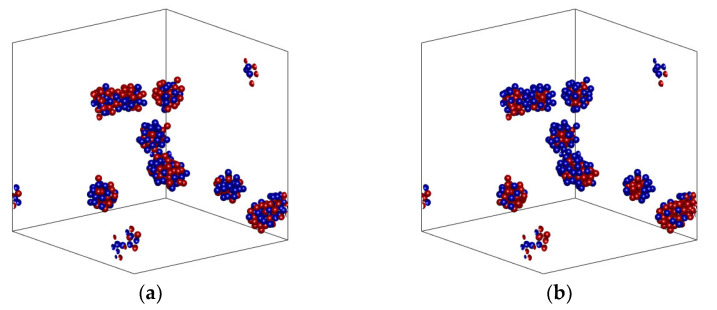

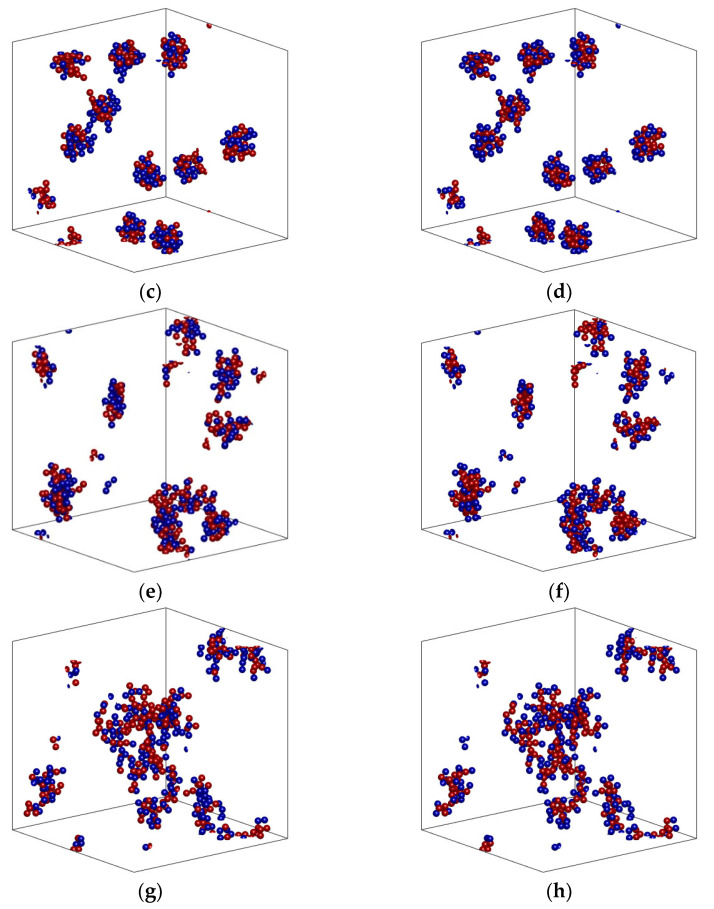
Aggregate configurations in a hybrid nanofluid, contained in a representative unit cell, 50% of particles with *k*_1_ conductivity, 42 particles per aggregate, total volume fraction 1%. (**a**,**c**,**e**,**g**) Random mixing configuration; (**b**,**d**,**f**,**h**) Core–Shell configuration (red: core, blue: shell) of the same particle positioning with corresponding Random mixing configuration on the left prior to particle labeling. (**a**,**b**) fractal dimension 2.8 (towards spherical form); (**c**,**d**) fractal dimension 2.5; (**e**,**f**) fractal dimension 2.2; (**g**,**h**) fractal dimension 1.9 (towards planar spread).

Figure 1a–h portray Random and Core–Shell configurations with equal populations of the particle types, for various fractal dimension values. The spatial distribution of the two particle types depends heavily on the shape of the aggregate. Specifically, spherical aggregates tend to have their core fully surrounded and shielded by the shell phase, with very few openings and pathways to the fluid phase. On the contrary, planar aggregates have particles of both phases in contact with the fluid phase. Shell particles tend to organize themselves in island-type regions at the edges of the planar configuration, with no clear connection path among all the shell particles of the aggregate. Conversely, core particles apparently do offer a continuous thermal path in the interior of a cluster and have many regions of contact with the bulk.

Depending on the type of particles that are placed in the core of the cluster, the terms “high Core–low Shell” and “low Core–high Shell” will be used in this work, with “low” and “high” referring to the corresponding lower or higher thermal conductivity of the two particle types.

This process can produce three sets of aggregate configurations, namely one of the Random type and two of the Core–Shell type (“high Core–low Shell” and “low Core–high Shell”). All three configurations have the same description macroscopically; however, their thermal behavior is expected to be different, depending on the spatial arrangement of the two types of particles. From a practical viewpoint, this is directly affected by the nanofluid preparation process and the different steps that are involved in it. The impact of the aggregate configuration features on the thermal conductivity of the hybrid nanofluid will be elucidated and discussed in this work, following a brief presentation of the numerical method for solving the heat transport equation in the aggregates and the base fluid.

## 3. Computation of Effective Conductivity

The geometry of the nanoaggregates as reconstructed by the algorithm of the previous section is imported in the heat transport simulations that are discussed in this section. A fixed temperature difference is imposed at the boundary faces perpendicular to an arbitrary direction, say *z*-, whereas periodic conditions are adopted on the lateral boundaries of the working domain. The Meshless Local Petrov–Galerkin (MLPG) method is used [32] for the numerical solution of the heat transport problem, since it has been shown to offer certain advantages over more conventional methods in particulate systems that have several single-contact points between objects, as is the present case. In the MLPG method, the differential equations are integrated locally, facilitating local resolution enhancement in regions of steep gradients. In addition to the abrupt thermal conductivity change across the interface between the nanoparticles and the base fluid, hybrid nanofluids also experience an abrupt conductivity change across contact points between particles of different type. This increases the difficulty for the reliable computation of the temperature profile in the working domain and the eventual computation of the effective conductivity, considering the increased number of nanoparticles of each type that must be placed in the working domain to ensure statistically adequate configurations. In fact, several realizations are also required for the same parameters given the random nature of the construction procedure. Cubic subdomains have been shown by the authors and co-workers to increase the stability of the MLPG method [31] and are used here. The DC PSE approach and the step functions are implemented as trial and test functions of the integration, accordingly [33,34]. Gauss quadrature is used for the calculation of the integrals [28,34]. The dimensionless weak formulation of the heat transport equation in any $\Omega_{s}$ subdomain is given by:

(3) $$\sum_{i}\left(k_{r,i}-1\right)\int_{\partial \Omega_{s}}\Phi_{i}{\nabla T}_{\hat{n}}d\left(\partial \Omega_{s}\right)+\int_{\partial \Omega_{s}}{\nabla T}_{\hat{n}}d\left(\partial \Omega_{s}\right)=0.$$

where $\Phi_{i}$ is the spatial step function, defined as unity in the interior of particulate phase *i* and zero elsewhere, and $k_{r,i}=\frac{k_{p,i}}{k_{f}}$ is the ratio of the conductivity of the particles of type *i* to that of the base fluid, i.e., the normalized thermal conductivity. The temperature profile throughout the computational domain is extracted from the solution of the heat transport equation. The effective conductivity of the nanofluid, *k_eff_*, is obtained from

(4) $$k_{eff}\frac{\Delta T}{\Delta z}=\bar{\int_{S}k\frac{\partial T}{\partial n}dS},$$

where the normal vectors of surfaces S are aligned with the macroscopic heat flow direction, *z*. More details on the numerical methodology, the relevant variables and integrals, the grid reconstruction, and the conductivity estimation can be found in previous works by the authors [29,34]. These works also include discussion of the corresponding predictions of analytical models for the effective thermal conductivity, including Maxwell [40] and Bruggeman [41] effective medium approaches. For hybrid nanofluids, the Maxwell 3-phase approximation of the effective conductivity should read as [42]

(5) $$k_{eff}=\frac{k_{0}\phi_{0}+\sum_{i\neq 0}k_{i}\phi_{i}\frac{3k_{0}}{2k_{0}+k_{i}}}{\phi_{0}+\sum_{i\neq 0}\phi_{i}\frac{3k_{0}}{2k_{0}+k_{i}}},$$

using subscript 0 for the base fluid, whereas *ϕ_i_* is the volume fraction of particle type *i* with conductivity *k_i_*. Bruggeman’s self-consistent three-phase EMT approximation of the effective conductance reads [43]

(6) $$\sum_{i}\phi_{i}\frac{k_{i}-k_{eff}}{k_{i}+2k_{eff}}=0.$$

Given that the above Effective Medium Approximations ignore the organization of nanoparticles into aggregates and consider the nanofluid as a fully dispersed system, a two-stage approach is also examined here, using the Bruggeman expression for each stage. The concept is similar to the one that was suggested in [44] for the prediction of the diffusivity in media containing core-in-shell-type inclusions. In the present case, the radius of gyration can be used to estimate the volume fraction within each aggregate, *ϕ_p_*, [45]:

(7) $$\phi_{p}=\left(\frac{R_{g}}{r_{p}}\right)d_{f}^{-3}.$$

This volume fraction is equal to the sum of the volume fractions of the two different constituents within the aggregates. In order to evaluate the individual volume fractions within the aggregates, as a first approximation, the ratio of the volume fractions at the scale of the entire nanofluid can be used at the scale of individual aggregates as well, unless some different information or data are available. At the next stage, the nanofluid is considered as a biphasic system, containing the carrier fluid and the homogenized aggregates with aggregate conductivity equal to the one that was calculated at the first stage, as described above. The volume fraction of the aggregates in the nanofluid, *ϕ_e_*, is calculated from the expression [29,46]

(8) $$\phi_{e}=\frac{\phi_{T}}{\phi_{p}}$$

where *ϕ_T_* is the total volume fraction in the nanofluid, *ϕ_T_* = *ϕ*_1_ + *ϕ*_2_. Equation (8) is easily derived from a simple mass balance at the two scales, namely that of the aggregates and that of the nanofluid.

## 4. Results and Discussion

To reduce statistical uncertainty, the simulation results presented here are extracted by averaging the conductivity results from 10 random realizations that are reconstructed with the same morphological features but using a different sequence of random numbers. Typically, a single hybrid nanofluid realization with 10 aggregates that contain 42 nanoparticles per aggregate requires ~6 h for convergence on a computer with 12 cores at 2.1 GHz and 512 GB RAM.

**Figure 2:**
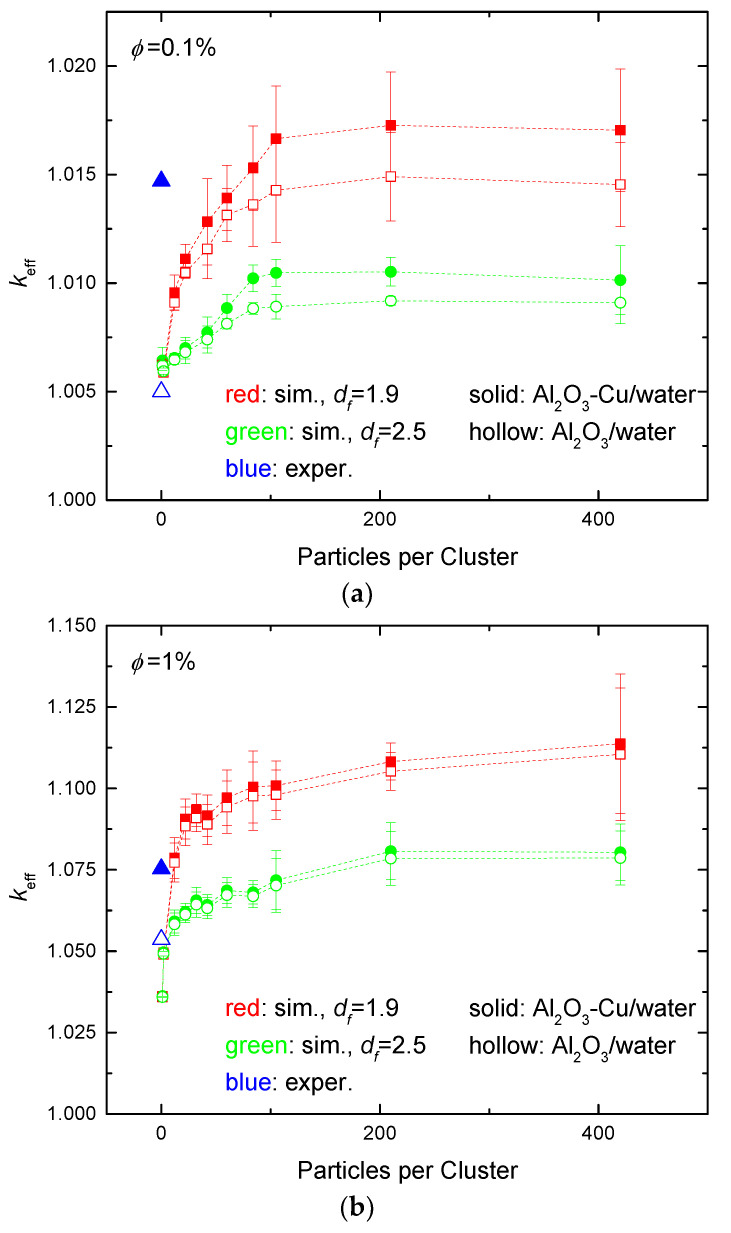

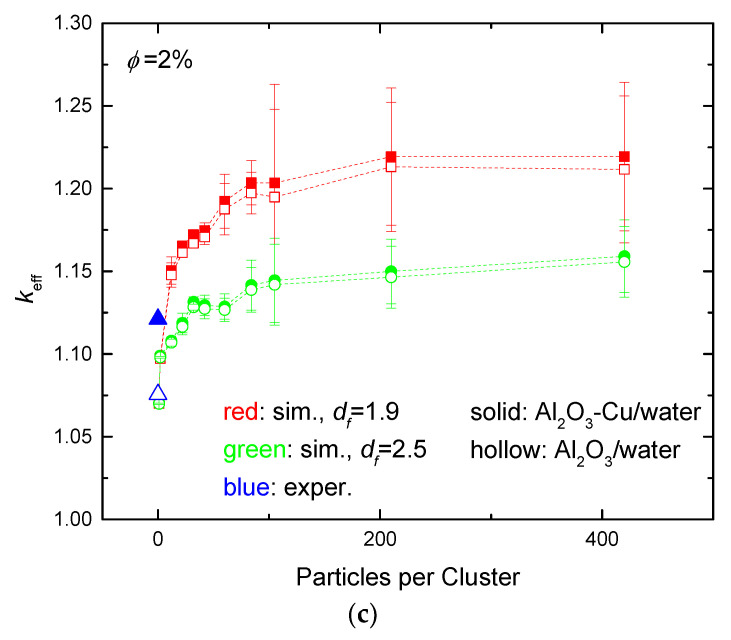
Normalized effective conductivity of Al_2_O_3_-Cu/water and Al_2_O_3_-water nanofluids as extracted from the method developed here and from experiments in literature [47], reproduced with permission from Elsevier, 2011, for total volume fraction of particles (**a**) 0.1%, (**b**) 1%, and (**c**) 2%; volume fraction of *k*_1_ particles (Al_2_O_3_) in hybrid nanofluids 96.21%; fractal dimension of nanoaggregates in simulations 2.5 and 1.9. Random mixing of the two types of particles is assumed.

First, some simulations are carried out to make the comparison with experimental observations regarding the effect of the aggregation level on the effective thermal conductivity of hybrid nanofluids, along with respective single-particle-type nanofluids. Suresh et al. [47] studied the thermal conductivity of an Al_2_O_3_-Cu/water hybrid nanofluid, along with an Al_2_O_3_/water simple nanofluid. They applied an Al_2_O_3_:CuO 90:10% mass ratio of particulates in the precursor batch. The nanoparticles appear to be practically of spherical shape, according to SEM images. Using the thermal conductivity values of 30, 398, and 0.611 W/m K for Al_2_O_3_ [48], Cu [49], and water [47], respectively, for the temperature of the experiments (32 °C), simulations of the thermal efficiencies of the Al_2_O_3_-Cu/water hybrid and Al_2_O_3_/water single nanofluids were performed in the present work using the proposed simulation methodology. Figure 2 presents the simulation results of Al_2_O_3_-Cu/water and Al_2_O_3_/water nanofluids for total particulate volume functions of 0.1%, 1%, and 2%, and a wide range of aggregation levels, namely from no aggregation/single particles, to heavy aggregation (420 particles per aggregate). The aggregates are reconstructed to acquire nearly spherical shape (fractal dimension *d_f_* = 2.5) or nearly planar configuration (*d_f_* = 1.9). The simulations reproduce the experimentally obtained effective thermal conductivity values of both hybrid and single nanofluid configurations and various aggregation levels and aggregation sphericity. The results show that, at low nanoparticulate loadings (*ϕ* = 0.1%), shown in Figure 2a, the thermal conductivity is compatible with highly dispersed Al_2_O_3_ in water, i.e., in the form of solitary particles, whereas Al_2_O_3_-Cu complexes correspond to a mildly aggregated (~50 particles per aggregate) configuration of planar clusters. For a moderate loading (*ϕ* = 1%), the simulation results indicate a weakly aggregated structure (~5 particles per aggregate) of Al_2_O_3_ in water, as expected, whereas for the Al_2_O_3_-Cu/water hybrid nanofluid, the effective thermal conductivity compares reasonably well with that of a more strongly aggregated spherical-like structure (~60 particles per aggregate). For elevated loading (*ϕ* = 2%), the thermal conductivities match well for moderately to strongly aggregated structures (more than ~25 particles per aggregate). In all cases, the simulations show that potential organization of nanoparticles in almost planar configurations would result in considerably increased thermal conductivity of the nanofluid, owing to the creation of long pathways across the working domain that are made up of nanoparticles with conductivity significantly higher than that of the carrier fluid. It is evident that if more information is available regarding the aggregate shape while suspended (and not sedimented), the current simulation approach can directly reproduce the actual particle configuration and provide estimates of the effective thermal conductivity value.

**Figure 3:**
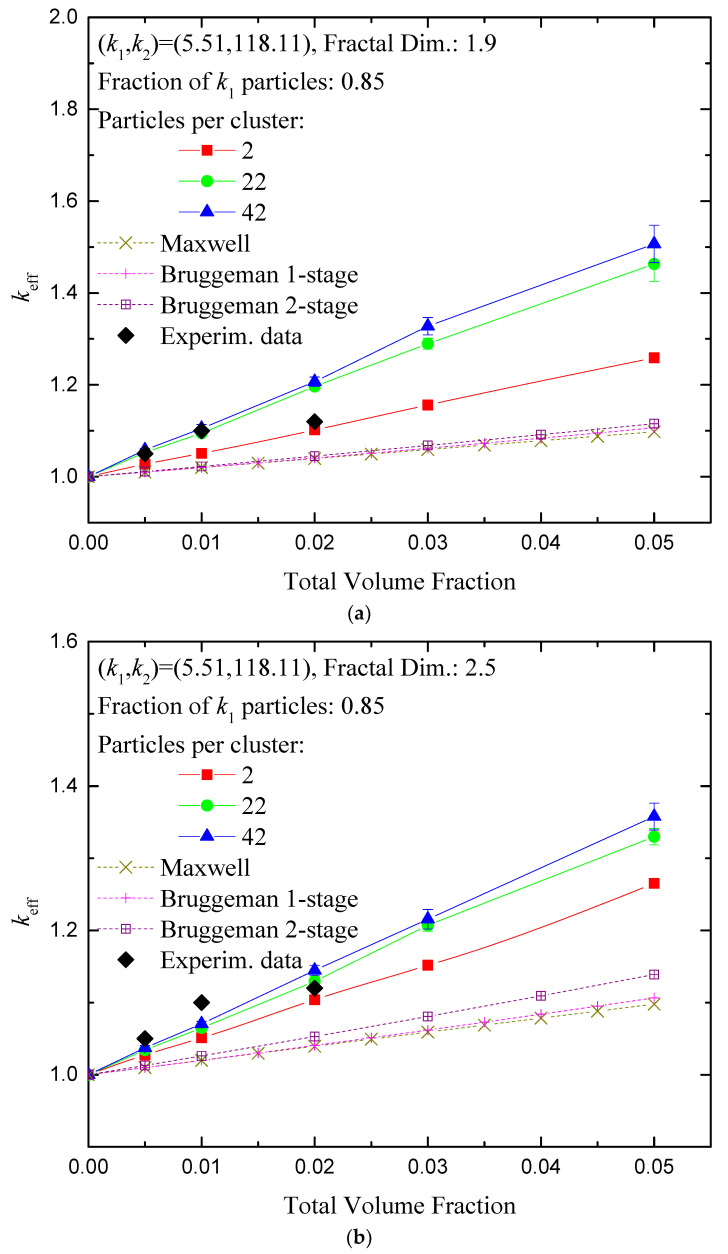
Normalized effective thermal conductivity of a hybrid nanofluid as a function of the total volume fraction of the two constituents (Random mixing), for 85:15 ratio of SiO_2_:MWCNT particles in EG. Experimental data extracted from [50], reproduced with permission from SNCSC, 2016, with no reference to fractal dimension. (**a**) Fractal dimension 1.9 (almost planar spread), (**b**) fractal dimension 2.5 (nearly spherical shape). Variation with the number of particles per cluster. The predictions of effective medium approximations are also shown.

A range of aggregate levels and typical fractal dimensions is also implemented in the present simulations to facilitate the comparison with other experimental data, such as the ones reported in [50], also reviewed in [11]. The effect of the total volume fraction of the nanomaterials in a hybrid nanofluid on the effective conductivity is shown in Figure 3a,b. Specifically, the case of SiO_2_:MWCNT particles in EG, i.e., (*k*_1_, *k*_2_) = (5.52, 118.11), homogeneously mixed at a 85:15 population ratio, is examined. Two characteristic fractal dimensions are shown, one towards planar spread (1.9), and one towards spherical configuration (2.5), for various numbers of particles per cluster. Comparison of the simulation results with experimental data is satisfactory for moderate numbers of particles per cluster, as seen in Figure 3. 

The predictions of the Maxwell and Bruggeman effective medium expressions for thermal conductivity as a function of the volume fraction of hybrid nanofluids, Equations (5) and (6), deviate significantly from the ones that were numerically computed here, showing a dependence that is much weaker than the one revealed by simulations. A similar observation was made for the comparison of the effective medium approximation with experimental data [51]. If the two-step Bruggeman approach is followed, as suggested in Section 3 of the present work, slightly improved predictions are obtained that now become sensitive to the existence of aggregates. However, as shown in Figure 3, even the two-stage effective medium approximation predicts a weaker dependence on the volume fraction than the one estimated by the numerical simulations. This is attributed to the fact that the effective medium approximation does not account for particle contact and local thermal bridging but rather assumes fully dispersed particles surrounded fully by the carrier fluid. Resorting to more complicated expressions that take into account the aspect ratio of the aggregates [52], which is clearly larger than one by simple inspection of configurations in Figure 1, has shown not to improve essentially the effective medium predictions. 

**Figure 4:**
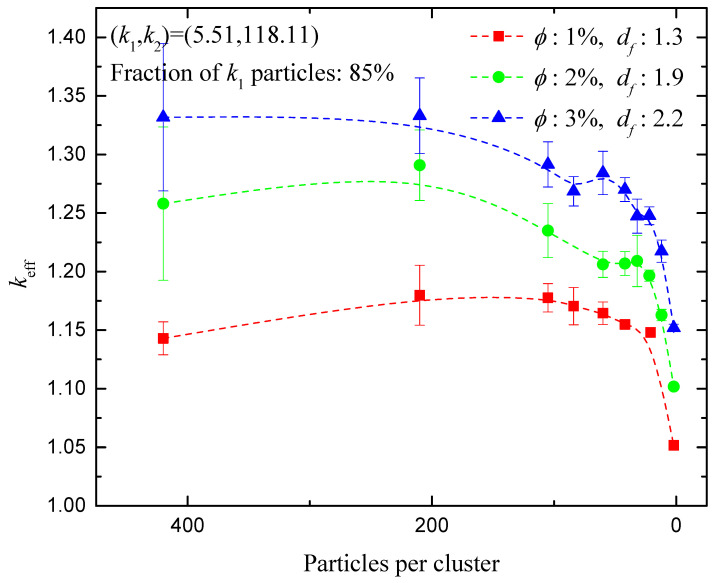
Normalized effective conductivity of a SiO_2_–MWCNT(85:15)/EG hybrid nanofluid as extracted using the method developed here, for fraction of *k*_1_ particles 85%, and characteristic configurations of total volume fraction (*ϕ*) and fractal dimension (*d_f_*) of nanoaggregates. Random mixing of particles is used.

Figure 4 shows in more detail the effect of the number of nanoparticles per aggregate on the effective conductivity of a hybrid nanofluid as obtained by the present simulator. The particles are distributed uniformly within each aggregate (Random mixing type). The ratio of 85:15 was used for the volume fractions of the SiO_2_–MWCNT mixture in ethylene glycol (EG). 

The maxima that are observed here are a clear indication of the interplay between the length of fast conduction pathways (within the aggregate volume) and distance between adjacent aggregates (slow conduction pathways within the carrier fluid). Strong agglomeration of the nanoparticles is evidently not the best state to increase heat transfer, nor is that of fully separated particles. This could be practical guidance for the design of the nanofluid preparation process. The same holds for applications that involve composite materials that contain nanoinclusions at different stages of particle organizations or, if pertinent, sintering level. This observation is in accord with that made in other literature [51] with the help of sonication, which is known to affect the clustering of particles. Specifically, the impact of sonication duration, along with that of other factors such as the existence of surfactants, on the thermal conductivity of a hybrid nanofluid (CeO_2_-MWCNT/water) was investigated in [53]. An optimal sonication time to reach maximum thermal conductivity was found [11], which is in line with the observation made here, namely that there exists some optimal value of the aggregation level to achieve maximum thermal conductivity.

**Figure 5:**
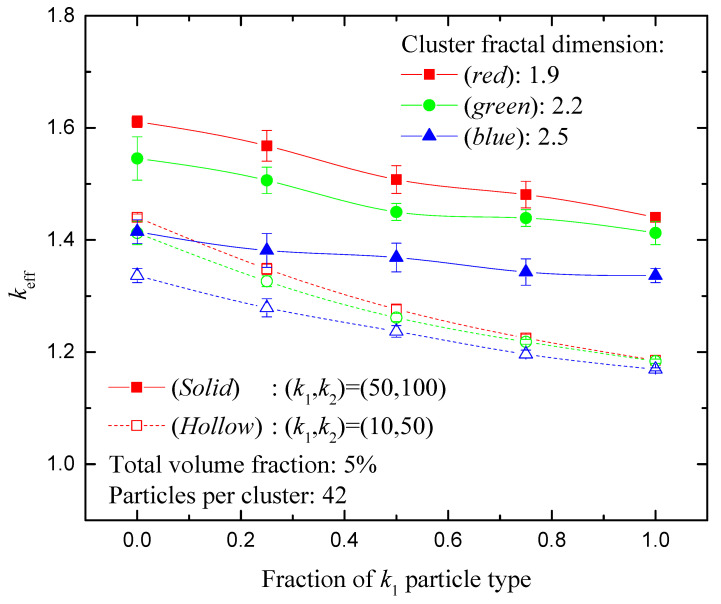
Normalized effective thermal conductivity of a hybrid nanofluid as a function of the mixing ratio of the two constituents (Random mixing), for 5% total volume fraction and 42 particles per cluster. Variation with the (*k*_1_, *k*_2_) pair of values, and the aggregate fractal dimension.

The mixing ratio of the two particle types in a hybrid nanofluid can influence the thermophysical properties remarkably. This influence is evident in Figure 5, which presents the effective thermal conductivity of a hybrid nanofluid as a function of the mixing ratio of the two particle types. A rather elevated total volume fraction of 5% is used in this graph, in order for the thermal performance effects to be more pronounced and, hence, more transparent to the reader. The particles are considered homogeneously distributed in each aggregate (Random mixing type). Two sets of particles are examined: (i) (*k*_1_, *k*_2_) = (10, 50), with relatively low conductivity values compared to that of the carrier fluid and relatively high *k*_2_/*k*_1_ ratio (5:1), and (ii) (*k*_1_, *k*_2_) = (50, 100), with relatively high absolute *k* values and relatively low *k*_2_/*k*_1_ ratio (2:1).

The monotonic dependence of the effective conductivity on the volume fraction of either particle type is shown in Figure 5, confirming the benefit of using a hybrid nanofluid to achieve improved thermal performance. It is noteworthy that this dependence is close to linear over, practically, the entire range of population ratio values. However, it must be noted that in the particular case of almost sphere-like clusters (*d_f_* = 2.5), the effective conductivity does not decrease considerably upon replacement of the fast-conducting particles with the relatively slow conducting ones. This can be attributed to the role of the fast-conducting particles that facilitate heat conduction through the nanofluid (thermal bridging). This suggests a concrete advantage of hybrid nanofluids over single-type ones: although the maximum thermal conductivity is, naturally, achieved in the absence of the less-conducting particles (fraction of *k*_1_ particles equal to zero), a close to maximum value is retained even in the (weak) presence of them in place of the more conducting ones.

In order to study the effect of the type of cluster configuration on the nanofluid conductivity, three types of cluster configuration are examined next, namely Random mixing, high Core–low Shell mixing, and low Core–high Shell mixing. For each configuration, 10 different realizations were considered, keeping the nanofluid data the same as those of the previous analysis, with 42 particles per cluster and a total volume fraction of 5%. The range of cluster fractal dimension varied between 2.8, corresponding to nearly spherical formation, and 1.9, indicating almost planar formation, in order to study the effect of the macroscopic cluster shape (both Random and Core–Shell), on the thermal performance expressed as the effective conductivity. 

**Figure 6:**
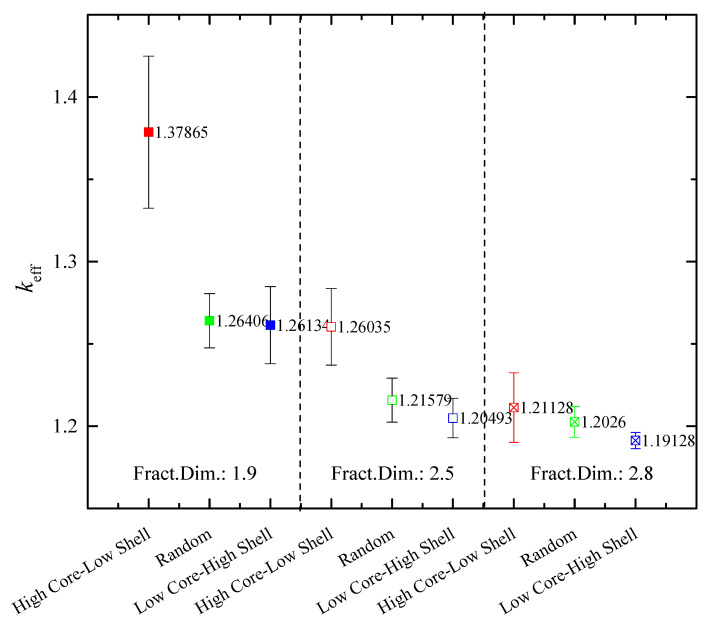
Normalized effective conductivity of a SiO_2_–MWCNT(50:50)/EG hybrid nanofluid for different configurations: Random, low Core–high Shell, and high Core–low Shell, for various cluster fractal dimensions.

Figure 6 displays the effect of the Core–Shell configuration on the performance of a SiO_2_–MWCNT(50:50)/EG hybrid nanofluid. For all types of particle spread in the aggregates, the high Core–low Shell configuration is found to be preferable for improved performance. This behavior can be attributed to the thermal bringing of the highly conducting particles within the cores, which is relayed, even at a lower rate, by the less-conducting shell particles. A similar argument explains the increased performance behavior of the high Core–low Shell configuration in the low fractal dimension case, i.e., more planar aggregates. Specifically, for *d_f_* = 1.9, the low-conductivity shell particles are sparse and isolated, and allow connection of the fast-conducting core particles with the base fluid and with other fast-conducting particles belonging to adjacent aggregates. In other words, the fast-conducting core particles are “widely” spread and form elongated thermal connectivity pathways, resulting in a significant increase in the effective conductivity compared to the low Core–high Shell and Random cases.

As expected, the opposite effect is noticed for low Core–high Shell configurations. Low-conducting nanoparticles are isolated within the core of the aggregates and have limited access to the base fluid and, consequently, limited contribution to conduction, while the fast-conducting shell nanoparticles cannot form fully connected conduction pathways. As a general conclusion, it can be stated that increased thermal conductivity is likely to obtain if the preparation of the nanofluid involves the addition of the high-conductivity particles first, followed by the dispersion of the low-conductivity particles in a second stage.

The fractal dimension has an even more pronounced effect on the enhancement of the effective conductivity, as shown in Figure 3. Specifically, switching from *d_f_* = 2.8 (nearly spherical clusters) to *d_f_* = 1.9 (nearly planar clusters) leads to almost double enhancement of the thermal conductivity even for a low volume fraction of particles (*ϕ* = 0.02). This is clearly attributed to the formation of long, almost continuous pathways for heat transfer across large regions of the nanofluid (see Figure 1).

## 5. Conclusions

In the present paper, the effect of adding a second particle type in a nanofluid on the thermal conductivity is studied in connection with aggregation phenomena. A method for reconstructing aggregates with a desired mixing ratio of two particle types is developed, reproducing major morphological characteristics of the aggregate, namely the fractal dimension, the number of particles per aggregate, and the spatial distribution of the particulate phases. A sophisticated MLPG meshless method with local refinement is used for the solution of the heat transport equation and for the calculation of the thermal performance of the hybrid nanofluid. The method is particularly stable for the complex particulate systems that are studied here. Adaptation and application of this method to hybrid nanofluid problems were of vital importance and allowed treatment of relatively large working domains that contain numerous particles at point contact with each other, thus facilitating the extraction of statistically meaningful results. Specifically, it was made possible to consider sets of 10 realizations of the hybrid configurations, containing up to 210 clusters and up to 420 particles in each realization.

The effective thermal conductivity for several cluster configurations and with different thermal conductivities of the constituent particles was estimated and compared with experiments from the literature. The present method involves a substantial extension of the aggregation algorithm that was presented in previous work by the authors [28,29], which was modified accordingly and adapted to the hybrid nanofluid case. The key advantage of this simulation procedure is that it allows the stochastic reconstruction of a complex dual system of particles suspended in a base fluid, capable of reproducing the desirable characteristics of the particulate system. The essential characteristics reproduced here include fractal dimension, volume fraction of the two types of particles, and sequential or random aggregation of the two types, combined with broad flexibility regarding the sequence of steps for the production of nanofluids with enhanced thermal properties. This reconstruction procedure yields similar morphologies to those produced by more conventional algorithms, like DLA, at greatly reduced computational times and resources, retaining the stochastic nature of sequential deposition and clustering at every step of the process.

The variation of the effective thermal conductivity was investigated over a wide range of fractal dimension values, the number of particles per aggregate, and the volume fractions of the two particle types. Compared to fully dispersed particles, aggregation was shown to increase the thermal conductivity up to a level, in the cases studied here. Further increase in the number of particles per aggregate appears to leave the conductivity approximately unaffected, but eventually reduces it for highly populated clusters. This result is qualitatively confirmed by experimental measurements [53], according to which nanofluids are processed by hyper-sonication to control the level of aggregation.

Both the Maxwell and the Bruggeman effective medium approaches of a hybrid nanofluid predict a monotonic increase in the effective conductivity with increasing particle content but at a rate that is clearly lower compared to the simulation results. A slight improvement is achieved if the two-stage Bruggeman approximation is followed, as described here, involving the calculation of the effective conductivity of the aggregates first, followed by effective medium calculations at the nanofluid scale, considered as a biphasic system at this second stage. The general conclusion is that the effective medium expressions appear to underestimate significantly the effect of particle aggregation on the nanofluid conductivity, mainly due to the fact that they ignore the organization of particles into clusters and their thermal bridging that is held responsible for the considerable increase in conductivity at the scale of individual aggregates.

The present study provides quantitative indications that the conditions of the production and dispersion of nanoparticles have a major impact on the thermal properties of hybrid nanofluids. The spatial distribution of the two particle types within the aggregates, in conjunction with the shape of the aggregate as described by its fractal dimension, are very important factors for the performance of the final nanosystem. All aggregate-type clusters were found to benefit considerably if made up of a high-conduction core and a low-conduction shell arrangement of particles that could be produced in a sequential particle addition manner in this order rather than mixing in a single step. Further, it was quantified how lowering the fractal dimension (that is, departing from spherical shape for the aggregates) favors the increase in thermal conductivity, mainly due to the creation of long continuous pathways across the nanofluid. Future efforts on the design and fabrication of hybrid nanofluids could benefit from these observations and adjust the preparation steps accordingly if the thermal conductivity of the resulting nanofluid is significant for the intended application.

It is also important to stress that the present methodology can be easily extended to handle hybrid nanofluids of more than two particle types. In addition, the methodology is equally applicable to nanocomposite materials containing multiple types of particle inclusions that are organized in aggregates. The present approach can be employed in a straightforward manner to reproduce the internal structure of nanocomposite materials, simulate nanofiller aggregation and sintering, and predict thermal and mass transport properties that are typically very important for a multitude of practical applications. Extension of this line of work to study mass transport and separation phenomena in mixed matrix membranes is straightforward.

## Data Availability

The original contributions presented in the study are included in the article, further inquiries can be directed to the corresponding author.

## References

[1] Choi S.U., Eastman J.A. (1995). Enhancing Thermal Conductivity of Fluids with Nanoparticles.

[2] Wen D., Lin G., Vafaei S., Zhang K. (2009). Review of nanofluids for heat transfer applications. Particuology.

[3] Yu W., Xie H. (2012). A review on nanofluids: Preparation, stability mechanisms, and applications. J. Nanomater..

[4] Pawłowski R., Kiełbasiński K., Sobik P., Pawłowski B., Wita H., Konefał R., Auguścik M., Pajor-Świerzy A., Szałapak J., Krzemiński J. (2019). Obtaining of silver nanopowders by the thermal decomposition of fatty silver salts with various chain length. Mater. Res. Express.

[5] Odularu A.T. (2018). Metal Nanoparticles: Thermal Decomposition, Biomedicinal Applications to Cancer Treatment, and Future Perspectives. Bioinorg. Chem. Appl..

[6] Sander M., West R.H., Celnik M.S., Kraft M. (2009). A Detailed Model for the Sintering of Polydispersed Nanoparticle Agglomerates. Aerosol Sci. Technol..

[7] Eggersdorfer M.L., Kadau D., Herrmann H.J., Pratsinis S.E. (2012). Aggregate morphology evolution by sintering: Number and diameter of primary particles. J. Aerosol Sci..

[8] Schmid H.-J., Al-Zaitone B., Artelt C., Peukert W. (2006). Evolution of the fractal dimension for simultaneous coagulation and sintering. Chem. Eng. Sci..

[9] Artelt C., Schmid H.J., Peukert W. (2005). On the impact of accessible surface and surface energy on particle formation and growth from the vapour phase. J. Aerosol Sci..

[10] Koch W., Friedlander S.K. (1990). The effect of particle coalescence on the surface area of a coagulating aerosol. J. Colloid Interface Sci..

[11] Rashidi M.M., Nazari M.A., Mahariq I., Assad M.E.H., Ali M.E., Almuzaiqer R., Nuhait A., Murshid N. (2021). Thermophysical Properties of Hybrid Nanofluids and the Proposed Models: An Updated Comprehensive Study. Nanomaterials.

[12] Turcu R., Darabont A., Nan A., Aldea N., Macovei D., Bica D., Vekas L., Pana O., Soran M.L., Koos A.A. (2006). New polypyrrole-multiwall carbon nanotubes hybrid materials. J. Optoelectron. Adv. Mater..

[13] Sajid M.U., Ali H.M. (2018). Thermal conductivity of hybrid nanofluids: A critical review. Int. J. Heat Mass Transf..

[14] Sarkar J., Ghosh P., Adil A. (2015). A review on hybrid nanofluids: Recent research, development and applications. Renew. Sustain. Energy Rev..

[15] Hemmat Esfe M., Esfandeh S., Kamyab M.H., Ali H.M. (2020). Chapter 1—History and introduction. Hybrid Nanofluids for Convection Heat Transfer.

[16] Estellé P., Halelfadl S., Thierry M. (2015). Thermal conductivity of CNT water based nanofluids: Experimental trends and models overview. J. Therm. Eng..

[17] Kleinstreuer C., Feng Y. (2011). Experimental and theoretical studies of nanofluid thermal conductivity enhancement: A review. Nanoscale Res. Lett..

[18] Jang S.P., Choi S.U. (2004). Role of Brownian motion in the enhanced thermal conductivity of nanofluids. Appl. Phys. Lett..

[19] Koo J., Kleinstreuer C. (2004). A new thermal conductivity model for nanofluids. J. Nanoparticle Res..

[20] Lotfizadeh S., Matsoukas T. (2015). A continuum Maxwell theory for the thermal conductivity of clustered nanocolloids. J. Nanoparticle Res..

[21] Xuan Y., Li Q., Hu W. (2003). Aggregation structure and thermal conductivity of nanofluids. AIChE J..

[22] Sarafraz M.M., Tlili I., Tian Z., Bakouri M., Safaei M.R., Goodarzi M. (2019). Thermal Evaluation of Graphene Nanoplatelets Nanofluid in a Fast-Responding HP with the Potential Use in Solar Systems in Smart Cities. Appl. Sci..

[23] Karagiannakis N.P., Skouras E.D., Burganos V.N. (2020). Modelling Thermal Conduction in Nanoparticle Aggregates in the Presence of Surfactants. Nanomaterials.

[24] Karagiannakis N.P., Skouras E.D., Burganos V.N. (2022). Modelling Thermal Conduction in Polydispersed and Sintered Nanoparticle Aggregates. Nanomaterials.

[25] Agista M.N., Guo K., Yu Z. (2018). A State-of-the-Art Review of Nanoparticles Application in Petroleum with a Focus on Enhanced Oil Recovery. Appl. Sci..

[26] Mahdi R.A., Mohammed H.A., Munisamy K.M., Saeid N.H. (2015). Review of convection heat transfer and fluid flow in porous media with nanofluid. Renew. Sustain. Energy Rev..

[27] Putnam S.A., Cahill D.G., Braun P.V., Ge Z., Shimmin R.G. (2006). Thermal conductivity of nanoparticle suspensions. J. Appl. Phys..

[28] Zhang X., Gu H., Fujii M. (2006). Experimental study on the effective thermal conductivity and thermal diffusivity of nanofluids. Int. J. Thermophys..

[29] Ghadimi A., Saidur R., Metselaar H.S.C. (2011). A review of nanofluid stability properties and characterization in stationary conditions. Int. J. Heat Mass Transf..

[30] Atluri S., Han Z., Rajendran A. (2004). A new implementation of the meshless finite volume method, through the MLPG “mixed” approach. CMES Comput. Model. Eng. Sci..

[31] Karagiannakis N.P., Bourantas G.C., Kalarakis A.N., Skouras E.D., Burganos V.N. (2016). Transient thermal conduction with variable conductivity using the Meshless Local Petrov–Galerkin method. Appl. Math. Comput..

[32] Wu X.-H., Tao W.-Q. (2008). Meshless method based on the local weak-forms for steady-state heat conduction problems. Int. J. Heat Mass Transf..

[33] Schrader B. (2011). Discretization-Corrected PSE Operators for Adaptive Multiresolution Particle Methods. Doctoral dissertation.

[34] Karagiannakis N.P., Bali N., Skouras E.D., Burganos V.N. (2020). An Efficient Meshless Numerical Method for Heat Conduction Studies in Particle Aggregates. Appl. Sci..

[35] Meakin P., Jullien R. (1988). The effects of restructuring on the geometry of clusters formed by diffusion-limited, ballistic, and reaction-limited cluster–cluster aggregation. J. Chem. Phys..

[36] Tence M., Chevalier J., Jullien R. (1986). On the measurement of the fractal dimension of aggregated particles by electron microscopy: Experimental method, corrections and comparison with numerical models. J. Phys..

[37] Koeylue U., Xing Y., Rosner D.E. (1995). Fractal morphology analysis of combustion-generated aggregates using angular light scattering and electron microscope images. Langmuir.

[38] Eggersdorfer M.L., Pratsinis S.E. (2012). The structure of agglomerates consisting of polydisperse particles. Aerosol Sci. Technol..

[39] Xiong C., Friedlander S. (2001). Morphological properties of atmospheric aerosol aggregates. Proc. Natl. Acad. Sci. USA.

[40] Torquato S., Haslach H.W. (2002). Random Heterogeneous Materials: Microstructure and Macroscopic Properties. Appl. Mech. Rev..

[41] Bruggeman D.A.G. (1935). Berechnung Verschiedener Physikalischer Konstanten von Heterogenen Substanzen. I. Dielektrizitätskonstanten und Leitfähigkeiten der Mischkörper aus Isotropen Substanzen. Ann. Physic..

[42] Brailsford A.D., Major K.G. (1964). The thermal conductivity of aggregates of several phases, including porous materials. Br. J. Appl. Phys..

[43] Wang J., Carson J.K., North M.F., Cleland D.J. (2008). A new structural model of effective thermal conductivity for heterogeneous materials with co-continuous phases. Int. J. Heat Mass Transf..

[44] Petsi A.J., Burganos V.N. (2012). Interphase layer effects on transport in mixed matrix membranes. J. Membr. Sci..

[45] Potanin A.A., De Rooij R., Van den Ende D., Mellema J. (1995). Microrheological modeling of weakly aggregated dispersions. J. Chem. Phys..

[46] Prasher R., Phelan P.E., Bhattacharya P. (2006). Effect of Aggregation Kinetics on the Thermal Conductivity of Nanoscale Colloidal Solutions (Nanofluid). Nano Lett..

[47] Suresh S., Venkitaraj K.P., Selvakumar P., Chandrasekar M. (2011). Synthesis of Al_2_O_3_–Cu/water hybrid nanofluids using two step method and its thermo physical properties. Colloids Surf. A Physicochem. Eng. Asp..

[48] Warlimont H., Martienssen W., Warlimont H. (2005). Ceramics. Springer Handbook of Condensed Matter and Materials Data.

[49] Touloukian Y.S., Powell R.W., Ho C.Y., Klemens P.G. (1970). Thermal conductivity—Metallic elements and alloys. (Reannouncement). Thermophysical Properties of Matter—The TPRC Data Series.

[50] Hemmat Esfe M., Behbahani P.M., Arani A.A.A., Sarlak M.R. (2017). Thermal conductivity enhancement of SiO_2_–MWCNT (85:15%)–EG hybrid nanofluids. J. Therm. Anal. Calorim..

[51] Yu W., France D.M., Routbort J.L., Choi S.U. (2008). Review and Comparison of Nanofluid Thermal Conductivity and Heat Transfer Enhancements. Heat Transf. Eng..

[52] Cherkasova A.S., Shan J.W. (2008). Particle Aspect-Ratio Effects on the Thermal Conductivity of Micro- and Nanoparticle Suspensions. J. Heat Transf..

[53] Tiwari A.K., Pandya N.S., Said Z., Öztop H.F., Abu-Hamdeh N. (2021). 4S consideration (synthesis, sonication, surfactant, stability) for the thermal conductivity of CeO_2_ with MWCNT and water based hybrid nanofluid: An experimental assessment. Colloids Surf. A Physicochem. Eng. Asp..

